# Supplementary material for: Effects of Particle Size of Curcumin Solid Dispersions on Bioavailability and Anti-Inflammatory Activities
Source: Antioxidants (Basel). 2023 Mar 15;12(3):724. doi: 10.3390/antiox12030724 (PMC10045274; doi:10.3390/antiox12030724)
Supplement: Supplementary file 1 [file antioxidants-12-00724-s001.zip › antioxidants-2268034-supplementary.pdf]

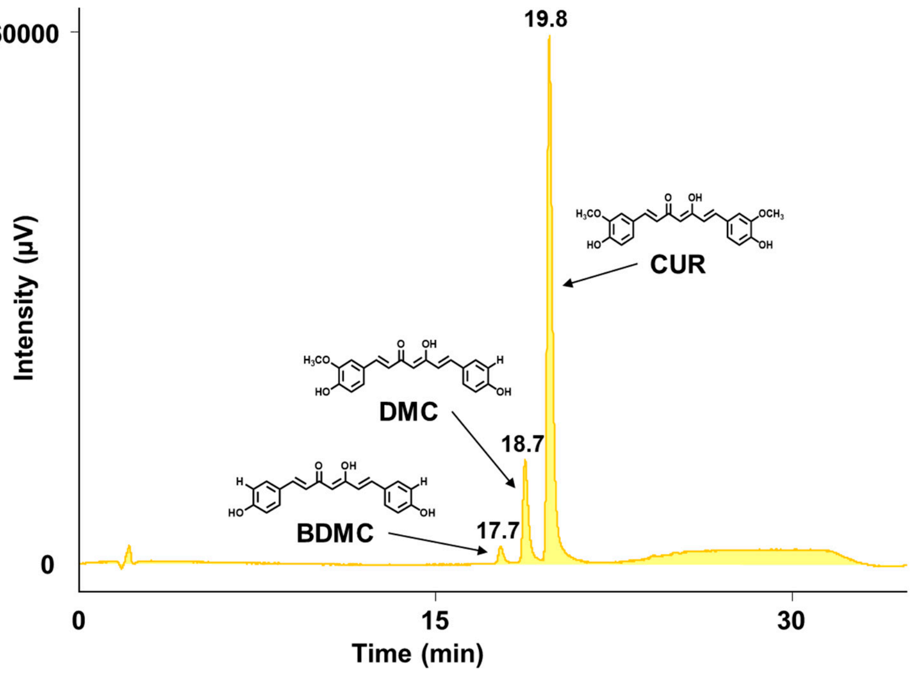


**Figure S1.** HPLC-UV chromatograms of C-SDs I.

CUR concentration of C-SDs I-IV were measured by HPLC-UV.

BDMC, bisdemethoxycurcumin; CUR, curcumin; C-SDs, curcumin solid dispersions; DMC, demethoxycurcumin.

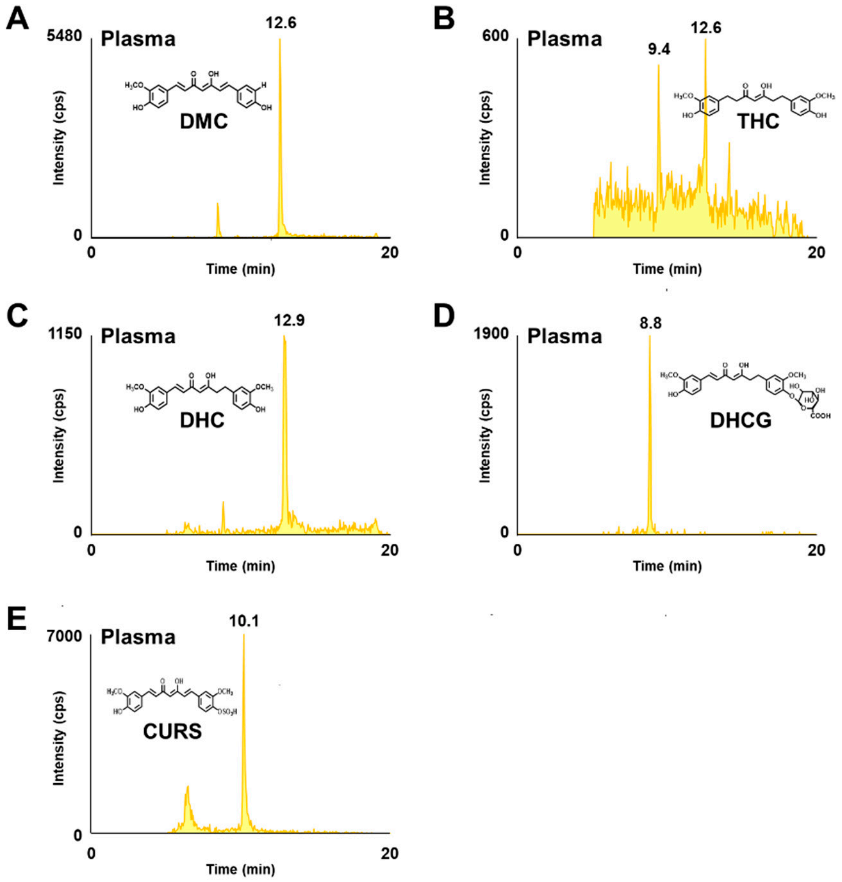


**Figure S2.** Representative HPLC-MS/MS chromatograms of other CUR metabolites in plasma.

Other CUR metabolites were analysed using MRM transitions from previous study [4], and DMC, DHC, DHCG, THC and CURS were detected in plasma.

CURS, curcumin sulfate; DHC, dihydrocurcumin; DHCG, dihydrocurcumin glucuronide; DMC, demethoxycurcumin; THC, tetrahydrocurcumin.

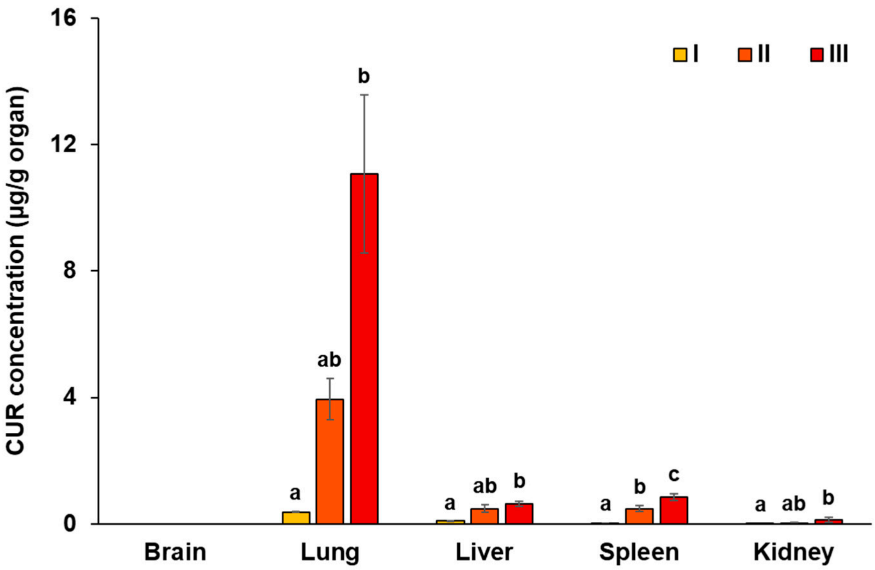


**Figure S3.** Organ distribution of CUR in the C-SDs I-IV intravenous administration groups.

Two hours after intravenous administration, organs (liver, spleen, lung, kidney, and brain) were immediately excised and rinsed with saline. Collected organs were stored at -80 °C until analysis. To prepare organ homogenates (25% w/v), each tissue was homogenized with a saline solution containing 1 mM ethylenediaminetetraacetic acid. 100 μL of tissue homogenates underwent extraction and quantification protocols as previously mentioned in section 2.4. (mean ± SE, n=4, a, b, c $P < 0.05$ (Tukey)).

I, C-SDs I; II, C-SDs II; III, C-SDs III.

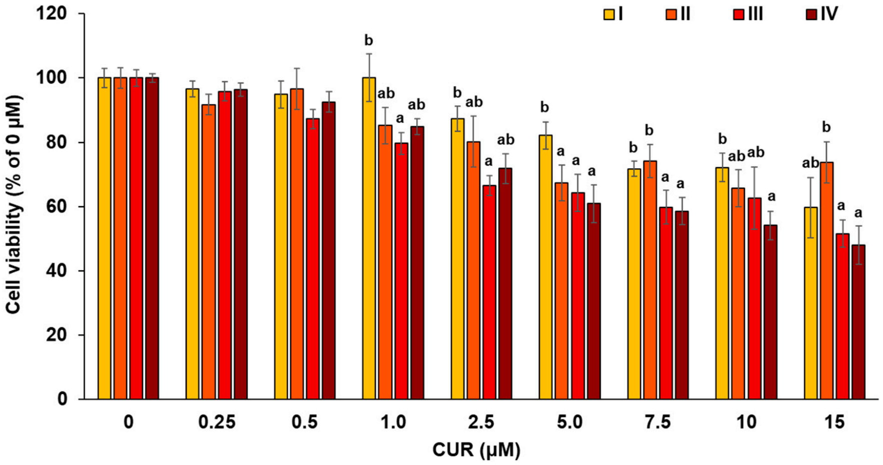


**Figure S4.** Cell viability

MTT assay was performed to determine cell viability based on the protocols described in the previous study [21]. Cell viability in LPS-stimulated RAW264 cells (positive control) is assumed to be 100%. (mean ± SE, n=6, a, b $P < 0.05$ (Tukey)).

MTT, 3-(4,5-dimethylthiazol-2-yl)-2,5-diphenyltetrazolium bromide; I, C-SDs I; II, C-SDs II; III, C-SDs III; IV, C-SDs IV.
